# Exploiting bacterial-origin immunostimulants for improved vaccination and immunotherapy: current insights and future directions

**DOI:** 10.1186/s13578-024-01207-7

**Published:** 2024-02-17

**Authors:** Guangyu Wang, Yongkang Wang, Fang Ma

**Affiliations:** 1 College of Food Science and Engineering, Collaborative Innovation Center for Modern Grain Circulation and Safety/Key Laboratory of Grains and Oils Quality Control and Processing, Nanjing University of Finance and Economics, Nanjing, Jiangsu 210023 China; 2 Institute of Veterinary Immunology & Engineering, National Research Center of Engineering and Technology for Veterinary Biologicals, Jiangsu Academy of Agricultural Sciences, Nanjing, 210014 China; 3 School of Food and Biological Engineering, Jiangsu University, Zhenjiang, 212013 China; 4 GuoTai (Taizhou) Center of Technology Innovation for Veterinary Biologicals, Taizhou, 225300 China

**Keywords:** Bacterial-origin immunostimulants, Vaccines adjuvants, PRR agonist, Innate immunity, Immunotherapy

## Abstract

Vaccination is a valid strategy to prevent and control newly emerging and reemerging infectious diseases in humans and animals. However, synthetic and recombinant antigens are poor immunogenic to stimulate efficient and protective host immune response. Immunostimulants are indispensable factors of vaccines, which can promote to trigger fast, robust, and long-lasting immune responses. Importantly, immunotherapy with immunostimulants is increasing proved to be an effective and promising treatment of cancer, which could enhance the function of the immune system against tumor cells. Pattern recognition receptors (PRRs) play vital roles in inflammation and are central to innate and adaptive immune responses. Toll-like receptors (TLRs)-targeting immunostimulants have become one of the hotspots in adjuvant research and cancer therapy. Bacterial-origin immunoreactive molecules are usually the ligands of PRRs, which could be fast recognized by PRRs and activate immune response to eliminate pathogens. Varieties of bacterial immunoreactive molecules and bacterial component-mimicking molecules have been successfully used in vaccines and clinical therapy so far. This work provides a comprehensive review of the development, current state, mechanisms, and applications of bacterial-origin immunostimulants. The exploration of bacterial immunoreactive molecules, along with their corresponding mechanisms, holds immense significance in deepening our understanding of bacterial pathogenicity and in the development of promising immunostimulants.

## Introduction

Vaccines could induce the host immune response to defense against infectious and noninfectious diseases, which is considered to be one of the most successful medical interventions. Since Edward Jenner’s landmark discovery in the late 18th century, vaccines have successfully reduced worldwide transmission levels to near eradication for some infectious diseases like hepatitis, malaria in humans, and rinderpest in animals [1, 2]. The limitations on vaccine efficacy often arise due to inadequate immunogenicity of certain antigens, resulting in insufficient immune responses. To address this challenge, researchers have turned to immune adjuvants as a way to enhance and modulate immune responses. Crucially, developing new vaccines with a rapid and efficient immune response stimulation is an urgent problem in combating the newly emerging diseases including novel coronavirus. Meanwhile, exploiting immune adjuvant to stimulate and regulate immune pathways can improve the effect of cancer immunotherapy.

**Fig. 1:**
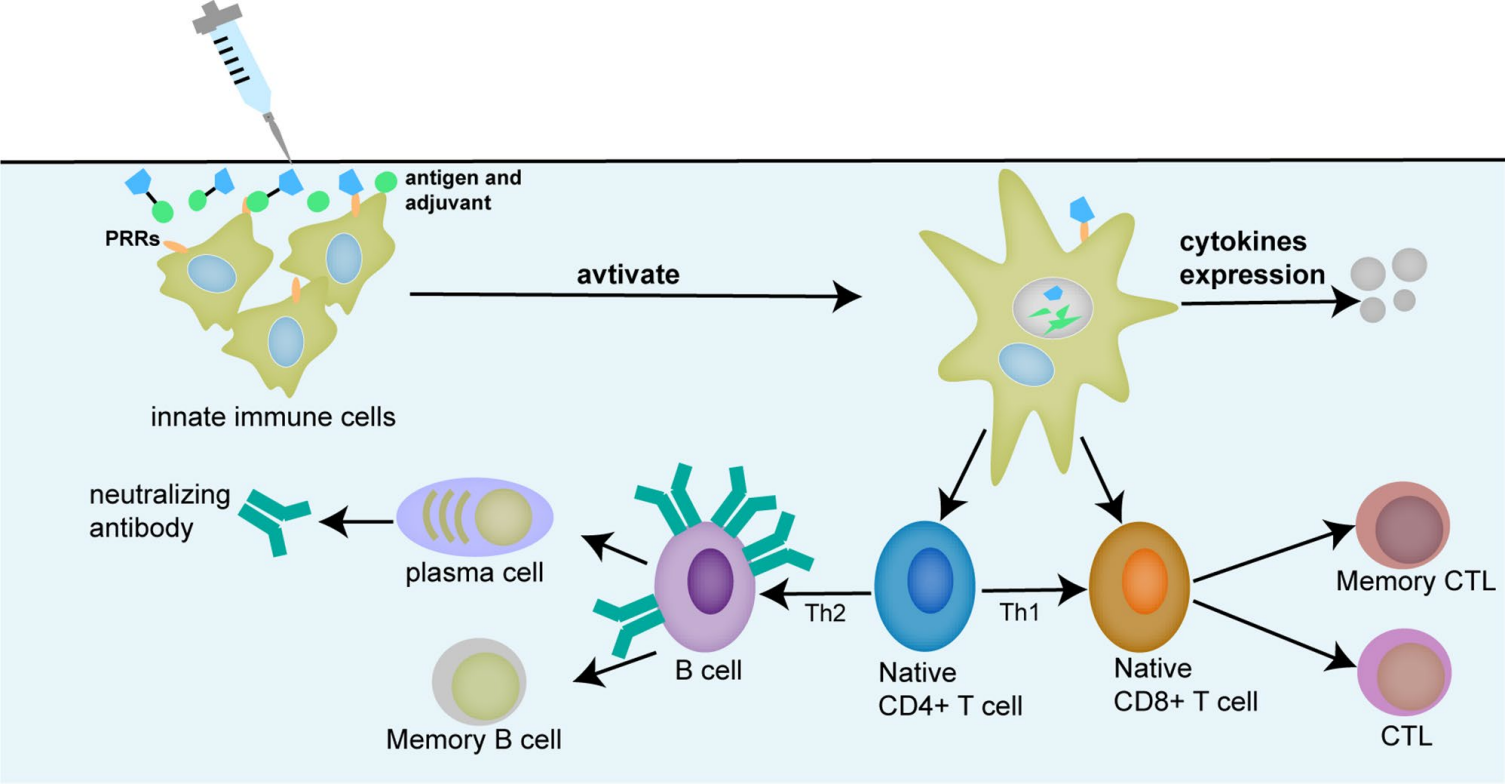
Schematic of immune response to the vaccine. Antigens are delivered to and bind with naïve dendritic cells to form mature antigen-presenting cells. Then the processed antigens bind with T-cell receptors on naïve CD4^+^ cells or naïve CD8^+^ cells, respectively. The stimulated T cells secrete cytokines to drive Th1 or Th2 immune response, leading to humoral or cellular immunity. In addition, activated dendritic cells can act directly on CD8^+^ T cells to license them to become CTLs. Adjuvants, mainly immunostimulants, interact with PRRs that induce the production of cytokines and chemokines, facilitating the generation of T helper cell response. CTL, cytotoxic T-lymphocyte

Immune adjuvants refer to substances that can change or enhance the immune response of the body to the antigen and enhance the immunogenicity of the corresponding antigen or change the type of immune response (Fig. 1). Adjuvants came to attention in the 1920s after Gaston Ramon, of the Pasteur Institute in France, made the crucial observation that inflammatory reaction at the site of injection using different substances, like tapioca and lanolin, enhanced immune response, and he had discovered the concept of adjuvant [3]. Immune adjuvants come from a wide range of sources, including aluminum salts, microorganisms and their derivatives, animal lipids, synthetic polymer materials, natural plant esters, polysaccharides, etc. Immune adjuvants can be categorized as immunostimulants and delivery systems [4]. The delivery system can promote antigen delivery by facilitating the uptake, transport, or presentation of antigens by antigen-presenting cells (APCs), however, immunostimulants can directly activate or enhance the host’s immune response [5]. Bacterial-origin immunostimulants are of particular interest due to their ability to directly activate or enhance the host’s immune response.

Many of the bacterial molecules trigger a more robust immune response by inducing affinity maturation of the antibody response, increasing serum antibody titers, and immunoglobulin class switching [6]. Bacterial active molecules are usually regarded as conserved pathogen-associated molecular patterns (PAMPs) for the activation of the innate immune system [7]. Stimulating the innate immune system is now known to have an important role in the evolution of the adaptive immune response [8]. Innate immune responses lead to a rapid burst of inflammatory cytokines and activation of APCs and then lead to the subsequent development of specific adaptive immune responses. The addition of such microbial PAMPs as immunostimulants leads to the development of robust and durable adaptive immune responses. Therefore, PAMPs, especially Toll-like receptor (TLR) ligands are considered attractive adjuvant candidates in vaccine development.

Both emergency infectious disease and cancer are major health threats worldwide. Immunostimulants play important roles in modern vaccine development, hence developing new materials to promote immune response against microbial infections and tumor cells has attracted the interest of multitudinous researchers. Some bacterial active molecules have been clinically applied, however, more efficient and safer bacterial-origin immunostimulants need to be discovered to promote and guide modern vaccine development. In this review, we will explore the current state of research on bacterial-origin immunostimulants and their potential impact on immunity.

## Bacterial active molecules

### Lipopolysaccharide (LPS) and monophosphoryl lipid A (MPL)

LPS is a complex molecule found in the outer membrane of Gram-negative bacteria that play a critical role in protecting the bacterial cell from environmental stresses. It was first described under the name of endotoxin by Pfeiffer as a highly toxic component firmly bound to cells of vibrio cholera [9]. TLR4 is a receptor that recognizes LPS on the surface of Gram-negative bacteria, triggering a signaling cascade that leads to the activation of the innate immune response. It forms a complex with MD-2, inducing myeloid differentiation 88 (MyD88)-dependent signaling and activating the NF-κB pathway. The activation of NF-κB eventually produces amounts of inflammatory cytokines, like interleukin (IL)-6, and tumor necrosis factor (TNF)-α [7]. However, it is clear that LPS also exacerbates the effects, which are known as major factors involved in the disastrous manifestations and clinical consequences of severe Gram-negative infections and generalized inflammation.

**Fig. 2:**
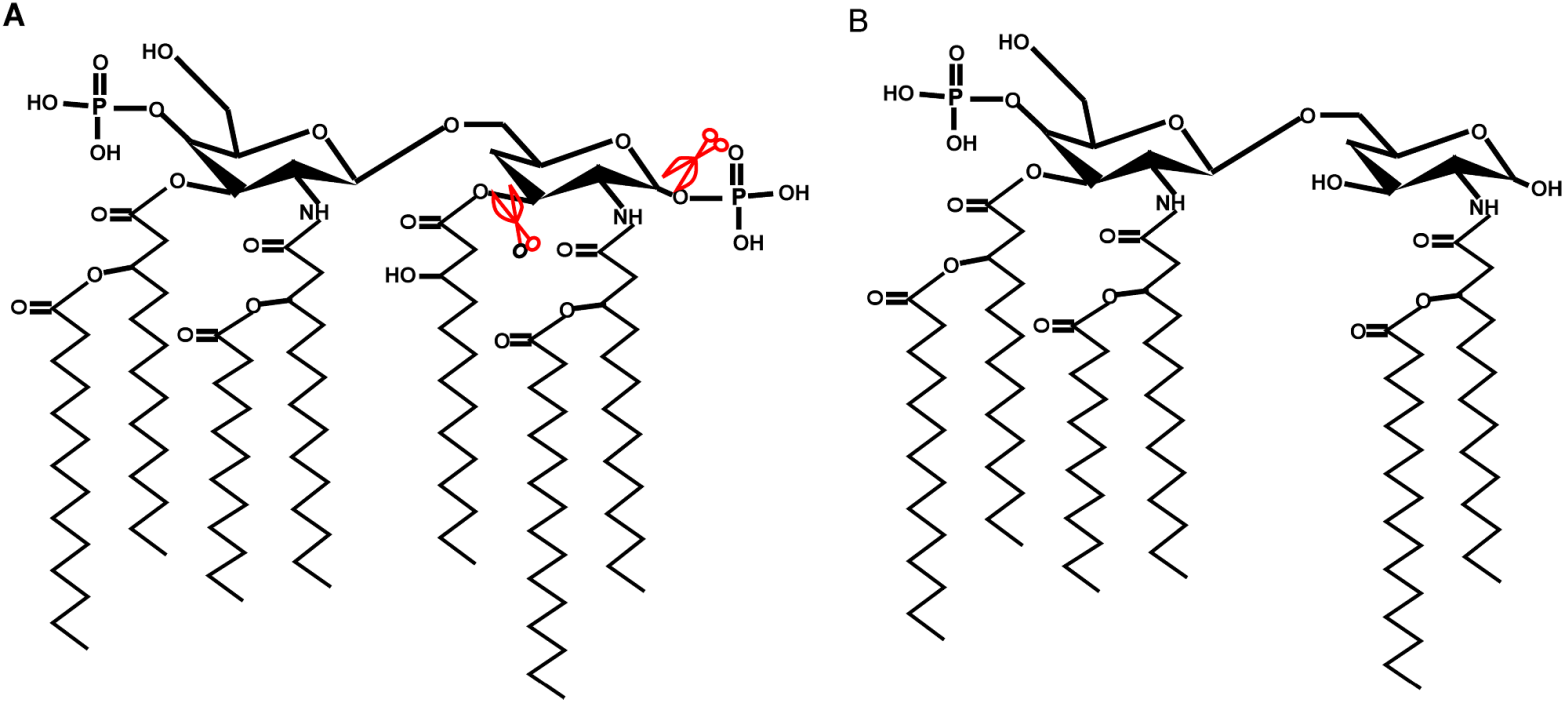
Structure of lipid A and MPL from *S. Minnesota* R595. (**A**) structure of lipid of LPS from *S. Minnesota* R595. (**B**) structure of MPL hydrolyzed from (**A**)

**Fig. 3:**
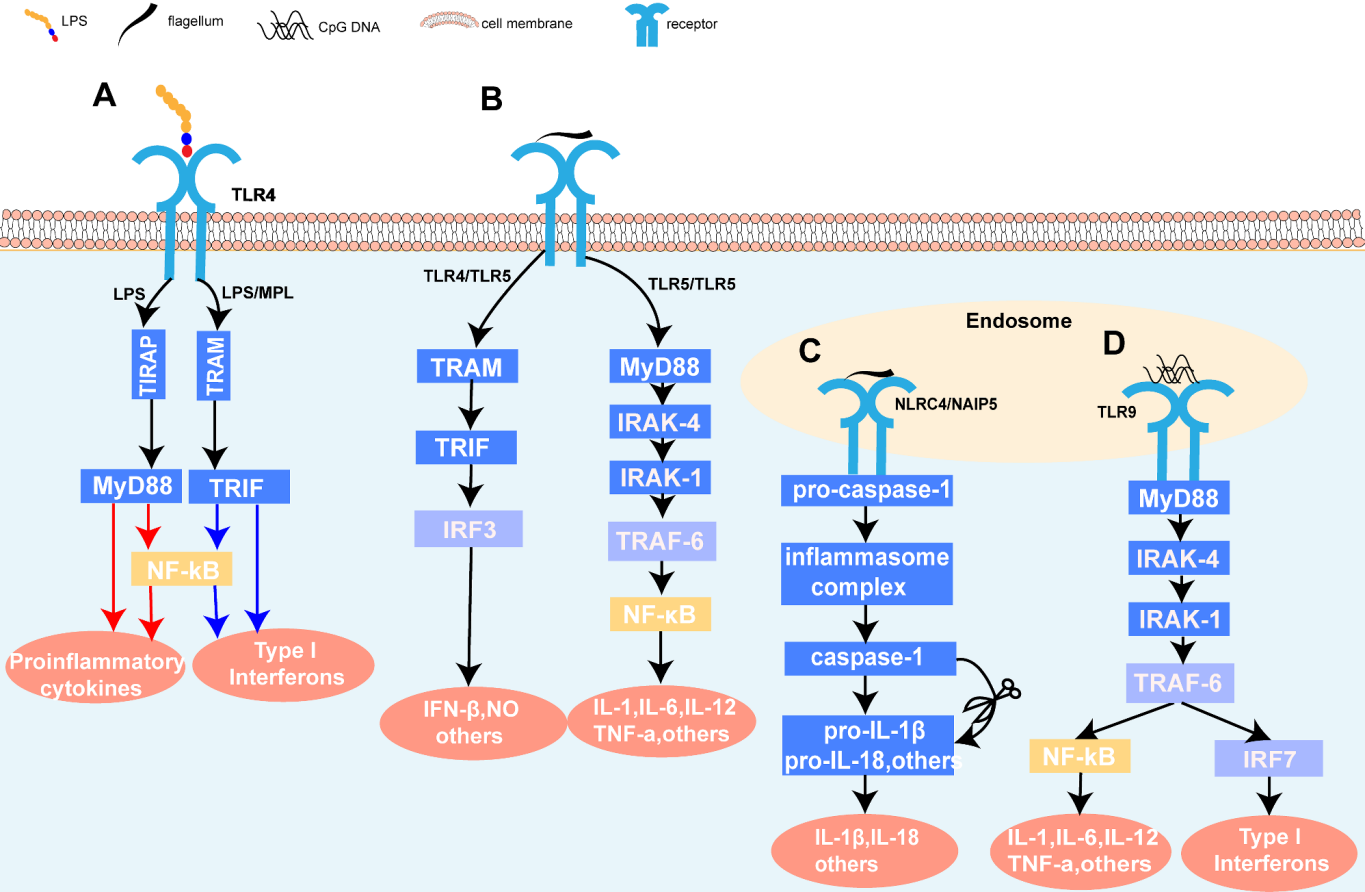
Schematic of representative bacterial-origin immunostimulants signaling pathway. (**A**) LPS and MPL interact with TLR4, recruiting its downstream adaptors. LPS induced both the MyD88-dependent and MyD88-independent signaling pathways, responsible for proinflammatory cytokine expression and Type I interferons, respectively. MPL activated the MyD88-independent signaling mediating the induction of Type I interferons. (**B**) The signaling cascade induction by flagellin can be through MyD88-dependent and MyD88-independent pathways. Flagellin activates the dimerization of TLR5 and then recruited MyD88 binding to the cytoplasmic portion of TLR5. The interaction leads to the activation of NF-κB signaling, inducing many downstream inflammatory-related genes expression. Meanwhile, TLR5 can form heteromeric complexes with TLR4 induced with flagellin, which lead to the activation of MyD88-independent signaling and production of NO. (**C**) Cytoplasmic flagellin activates caspase-1 and secretion of IL-1β via NLRC4/NAIP5-dependent inflammasome pathway. (**D**) CpG-ODN interacts with TLR9 in the endosome and recruits MyD88 and the downstream molecules, which activates either NF-κB or IRF7 according to the type of endosomes. The activation of NF-κB is responsible for the production of pro-inflammatory cytokines, like IL-1, IL-6, IL-12, and TNF-α, while the activation of IRF-7 is responsible for the expression of type I interferons. LPS, lipopolysaccharide; MPL, monophosphoryl lipid A; TLR, Toll-like receptors; MyD88, myeloid differentiation 88; NO, nitric oxide; IL, interleukin; NLRC4, Nod-like receptor protein 4; NAIP5, Nucleotide-binding domain leucine-rich repeat family, apoptosis inhibitory protein 5; CpG-ODN, CpG Oligodeoxynucleotides; IRF7, Interferon regulatory factor 7; TNF, tumor necrosis factor

LPS is composed of three major parts: an O-specific chain, core polysaccharide, and Lipid A, which is the toxic component / harbors the ‘endotoxic principle’ of LPS responsible for the pathophysiological effects associated with severe Gram-negative infections [10]. However, due to its toxicity, Lipid A cannot be used directly as an immunostimulant. MPL, a nontoxic derivative of LPS obtained by hydrolyzing Lipid A, retains the immunostimulatory properties of LPS but lacks many of its toxic effects, making it a promising adjuvant for vaccine development. MPL™ (Corixa) adjuvant is a 3-O-desacyl-4’-monophosphoryl lipid A is detoxified derivative of the LPS isolated from the Gram-negative bacterium *Salmonella Minnesota* (*S. Minnesota*) R595 strain [11]. LPS treated with acid hydrolysate was shown to be the 4’-monophosphoryl derivative of the lipid A moiety which is confirmed to be a potent immunostimulant and lacks many of the toxic properties of the parent LPS [12] (Fig. 2). Subsequent mild alkaline treatment of MPL selectively removes a single fatty acid, resulting in a further reduction in toxicity without diminishing its immunostimulatory efficacy [13]. Vaccine Adjuvant System (AS), specifically AS01, AS02, AS04, and AS15, incorporate MPL™ as an immunostimulatory ingredient. Importantly, AS04, which consists of MPL™ adsorbed onto aluminum salts, has received approval for incorporation in the human papillomavirus (HPV) vaccine (Cervarix) and hepatitis B virus (HBV) vaccine (Fendrix). The innate immune responses elicited by AS04 are attributed predominantly to MPL. This adjuvant stimulates robust antibody production, enhances Th1 cell responses, and increases the frequency of HPV-specific memory B cells [14]. Both LPS and MPL are recognized specifically by TLR4. However, MPL uniquely triggers signaling exclusively via the TRIF adaptor, whereas LPS activates TLR4 through both TRIF and MyD88 pathways. The MyD88 pathway resulting in high levels of many inflammatory cytokines, prominently TNF-α [15] (Fig. 3). MPL can stimulate cytokines and chemokines and initiate both innate and adaptive immune responses [16]. MPL also promotes the migration and maturation of antigen-presenting cells [17]. As a Th1-type adjuvant, MPL has shown a good safety profile in human and animal studies, making it a promising candidate for use in vaccines for global health. TLR4 agonists such as MPL can enable the development of new generation vaccines that are more effective at stimulating immune responses and protecting against infectious diseases and tumors.

### Bacterial outer membrane protein (OMP) and lipoprotein

All Gram-negative bacteria possess surface-associated OMPs, some of which have long been recognized as potential vaccine candidates. Purified preparations of meningococcal OMPs noncovalently complexed with antigens via hydrophobic interaction to form multimolecular vesicular structures, which were regarded as proteosomes. In clinical trials I and II, combinations of *Neisseria meningitidis* (*N. meningitidis*) OMPs-proteosome and *Shigella flexneri* 2a or *Pleisiomonas shigelloides* LPS (Protollin ™) were used as an intranasal adjuvant and shown to be safe and well-tolerated [18, 19].

Bacterial lipoproteins are secreted proteins anchored to the cell membrane, playing important roles in bacterial life activities [20]. The first lipoprotein in *E. coli* was described by Braun and Rehn in 1969, and the lipidation pattern at the N-terminus of bacterial lipoproteins, the cysteine-linked diacyl lipid portion, which acts as potent agonists of TLRs [21, 22]. For example, *E. coli* expressed bivalent recombinant LP2086, the second recombinant lipoprotein licensed for use in human vaccines, has been shown to prevent *N. meningitidis* serogroup B disease in children and adolescents [23]. Lipoprotein BP1569 of *Bordetella pertussis* (*B. pertussis*) has been shown to activate mouse dendritic cells (DCs), macrophages, and human monocytes. Subsequently, synthetic lipopeptide LP1569 was shown to enhance pertussis vaccine-induced Th1, Th17, and IgG2a antibody response against *B. pertussis* infection [24]. Synthetic lipopeptide of the N-terminal lipopentapeptide and lipohexapeptide Pam3-Cys-Ser-Lys4 (Pam3CSK4) of bacterial lipoprotein have also been shown to act as potent immunostimulants [25, 26].

However, the members of bacterial lipoprotein family are abundant and largely remains unclear. The diacyl-glyceryl and triacyl-glyceryl structures of lipoproteins could activate TLR2/TLR6 or TLR2/TLR1 signaling to induce innate immune responses [27, 28]. Based on these structures, lipoproteins are promising immunostimulants candidates, while they often play a role in pathogenicity in the context of an infection. Their lipopeptide analogues are synthesized and investigated for biological activity [29]. However, interaction between lipoproteins/lipopeptides and TLR2 induce most diverse of molecule signaling. Therefore, researches on the structure-activity relationship of immunostimulants are necessary and synthetic lipopeptides are the primary candidates, due to the diverse convenient chemical modification. Bacterial OMPs and lipoproteins are promising vaccines and immunostimulants candidates based on previous studies. However, due to the complexity and diversity of their structures, the biological activity and mechanism of these compounds remain uncertain and require further investigation.

### Flagellin

**Fig. 4:**
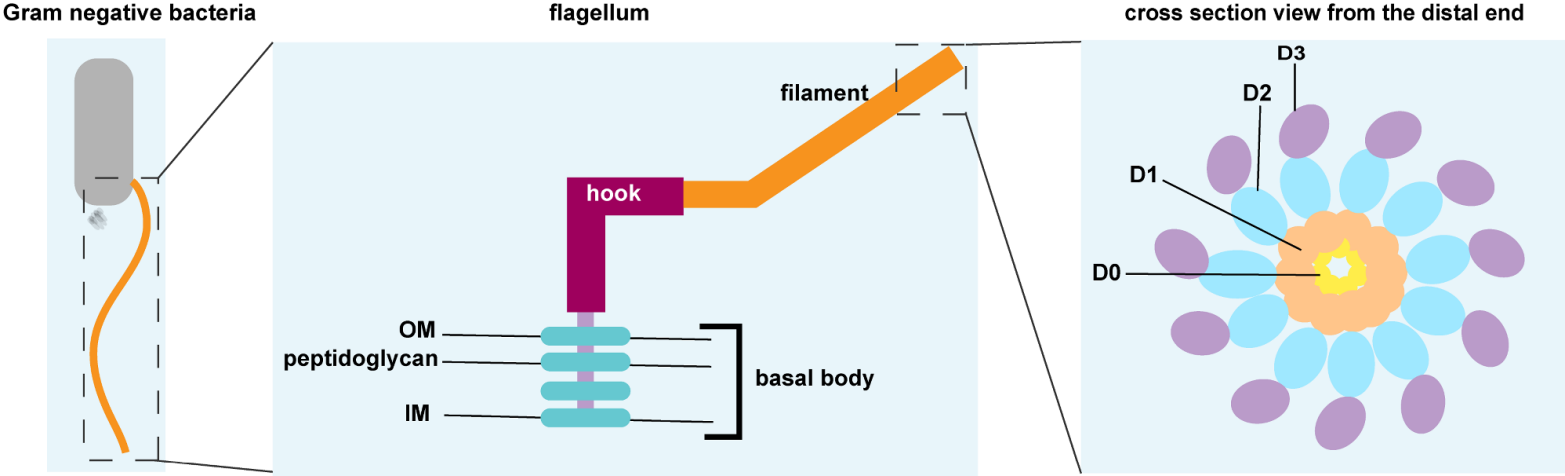
Structure schematic of the bacterial flagellum. The bacterial flagellum structure consists of a basal body, a hook, and a filament. The basal structure is anchored in the outer and inner membranes. The hook is external to the cell, lying between the basal body and the filament. Filament consists of four domains, D0, D1, D2, and D3, in end-on view. Antibody responses tend to be targeted to the filament

The flagellum is a long filamentous appendage on the surface of bacteria, which is composed of three parts: the basal body, the torsion hook, and a helical hollow filament [30]. The flagellar filament is composed of thousands of copies of the protein flagellin [31]. Flagellin acts as a potent immunostimulant, it engages the innate immune system by activating the extracellular TLR5 signaling pathway and intracellular Nod-like receptor protein 4 (NLRC4) signaling pathway [32, 33] (Fig. 3). Flagellin can be divided into four domains, labeled D0-D3, arranged from the inside to the outside of the filament [34] (Fig. 4). The adjuvant activity of flagellin was first reported by McEwen, and subsequent studies have demonstrated its effectiveness as an adjuvant, eliciting potent systemic and mucosal adaptive immune responses [35, 36]. The mucosal immune response serves as the first line of defense against many pathogens in the respiratory, gastrointestinal, and genitourinary tract due to the expression of TLR5 in mucous tissue [32, 37]. TLR5 recognizes only flagellin monomers and the recognition site is buried in the core of the flagellar filament [38]. This indicates that cellular recognition of flagellin requires breaking down of the filament to into its monomeric form. The crosstalk between flagellin and epithelial cells is essential for the recruitment of DCs. The recruitment of DCs into the epithelium is a prerequisite to initiate an adaptive response [39]. Taking advantage of TLR5- and NLRC4- stimulating activities, flagellin has been actively developed as a vaccine adjuvant and immunotherapeutic.

Flagellin can be mixed with vaccine antigens, chimerically expressed, or co-displayed with foreign antigens in live bacterial strains, incorporated into nanoparticles, and used as an adjunctive therapy for tumors. In addition, Vaccination with recombinant fusion antigens physically linked with flagellin has shown greater efficacy [40]. To date, four candidate vaccines based on flagellin-fusion technology have entered clinical trials, including a plague vaccine, recombinant M2e-flagellin influenza vaccine (STF2.4xM2e, VAX102), recombinant HA-flagellin influenza vaccine (STF2.HA1 SI, VAX125) and a prototypic quadrivalent seasonal HA-flagellin influenza vaccine (STF2, VAX2012Q) [41]. The plague vaccine, a recombinant flagellin-F1-V protein against *Yersinia pestis*, is evaluated in a phase I clinical trial [42]. VAX102, a recombinant of four copies of the ectodomain of M2e antigen and *Salmonella typhimurium* flagellin phase 2 (STF2), and phase I trials show a fourfold rise in anti-M2e antibodies in human [43]. VAX125 vaccine consists of the globular head of the HA1 domain of H1N1 fused with STF2 and has advanced to phase II study, making it a promising candidate for the prevention of influenza A disease in elderly patients [44]. VAX2012Q comprises four hemagglutinin subunits, each fused to a flagellin protein, and was studied in a phase I clinical trial. All four components of VAX2012Q elicited seroprotective immune responses against respective influenza type A or B viruses, and no safety concerns were reported [45]. Flagellin enhances antigen immunogenicity and induces both humoral and cellular responses against infectious pathogens, making it a promising tool for the development of novel vaccines. Although none of the flagellin-adjuvanted vaccines has received approval for sale or use, a flagellin-fusion influenza vaccine has advanced to phase II studies. Phase III studies of flagellin influenza vaccines are expected to be completed within the next five years. Flagellin-based nanoparticles and optimized derivatives are also rapidly developing in novel vaccine design and tumor immunotherapy. However, one potential concern is the ability of flagellin to induce anti-flagellin antibodies, which could potentially interfere with its adjuvant activity. Therefore, deeper understanding and optimization of flagellin derivatives would expedite clinical application.

### Bacterial CpG oligonucleotides

Tokunaga et al. found that *Mycobacterium bovis* (*M bovis*) strain Bacillus Calmette-Guérin (BCG) DNA possessed antitumor activity [46]. Six-base self-complementary ‘palindromes’ in microbial DNA, which are capable of immune stimulation were shown to contain at least one cytosine-phosphate-guanine (CpG) dinucleotide [47]. Synthetic oligodeoxynucleotides containing unmethylated CpG dinucleotide, flanked by two 5’ purines and two 3’ pyrimidines, could induce more than 95% of all spleen B cells to enter the cell cycle, suggesting the immune stimulation of bacterial DNA with non-methylated CpG motifs (CpG ODN) [48]. CpG motifs are unique to microbial DNA since vertebrate DNA is methylated at CpG sites, making it non-stimulatory. The immunostimulatory effects of bacterial DNA can be mimicked by synthetic oligodeoxynucleotides containing a CpG ODN. Several types of synthetic CpG ODNs have been identified, including A-class (D-type), B-class (K-type), C-class, and P-class (Table 1). Differences in retention times of CpG/TLR9 complexes in pDC endosomes can explain the distinct activities of different types [49]. A/D class CpG ODNs are characterized by high interferon-α (IFN-α) but intrinsically form aggregates, hindering their good manufacturing practice grade preparation [50]. The structure-function relationship of B-class CpG ODNs has been extensively investigated to enable their clinical use.

**Table 1:** Comparison of A, B, C, and P class CpG

class	Structure characteristics	Immunomodulatory activity	Reference
CpG-A	phosphodiester CpG motif(s) phosphorothioate-G-rich 3’ and 5’ ends	strong pDC IFN-α induction weak B cell activation.	[146, 147]
CpG-B	multiple CpG motifs on a phosphorothioate backbone	potent Th1 strong B cell and NK cell activation triger TNF-α and IL-6 production weak pDC IFN-α induction	[148]
CpG-C	wholly phosphorothioate no poly-G stretches palindromic sequence	good induction of IFN-α in pDCs activating B cells good Th1 induction	[149, 150]
CpG-P	two palindromic sequences form concatemers multimeric units	strong induction of IFN-α production	[151]

pDC, plasmacytoid dendritic cell; IFN, interferons; NK cell, natural killer cell; TNF, tumor necrosis factor; IL, interleukin

Halperin et al. first reported CpG used as the sole adjuvant and the phase I study showed the safety and immunogenicity of a 22-mer synthetic, phosphorothioate oligodeoxyribonucleotide immunostimulatory DNA sequences (CpG 1018) co-administered with hepatitis B surface antigen in healthy adults [51]. CpG 7909 as an adjuvant to Engerix-B (GlaxoSmithKline) HBV vaccine and pneumococcal conjugate vaccine in HIV-infected adults was confirmed in phase I/II study [52]. In addition, CpG ODNs are most studied the potential for cancer treatment in human clinical trials such as Non-Hodgkin’s lymphoma, renal cell carcinoma, melanoma, and non-small cell lung cancer, including ND1018 ISS of class B CpG ODN from Dynavax, IMO 2055 from Idera and agatolimod from Pfizer [53].

CpG ODN can induce a good Th1-type immune response via synergy between TLR9 and the B-cell receptor, resulting in antigen-specific B-cell stimulation, inhibition of B-cell apoptosis, enhanced IgG class switching and DC maturation and differentiation [48, 54–56]. CpG ODNs interact with TLR9 in the endosome, recruiting MyD88 and downstream signaling molecules to activate NF-κB and IRF-7, resulting in the production of inflammatory cytokines and type I IFNs [57] (Fig. 3). Additionally, Miguel et al. found that CPG ODNs activate a non-TLR9-dependent signaling pathway through the Src kinase family, triggering a tyrosine phosphorylation-mediated signaling cascade involved in upstream MyD88 signaling [58]. CpG ODN-antigen conjugates enhance immunogenicity by ensuring that both antigen and CpG ODN are taken up by the same APC and improving uptake on these cells, and this effect is independent of the nature of the ODN but requires physical conjugation of DNA to target antigen [59]. In addition, inclusion of CpG ODNs further enhances the utility of cholera toxin, a leading experimental mucosal adjuvant for the induction of strong mucosal immune responses [60]. Mucosal immunization with CpG ODN alone could also induce strong mucosal immune responses [61]. Currently, there are many ongoing or completed clinical trials studying B-class CpG ODNs as adjuvants targeting infectious agents, allergens, and cancer. CpG ODN-based therapies for allergens and cancers needs to be exploited further as an immunostimulant. Exploration of other and new classes of CpG ODNs could advance progress in the areas of immunity, chemistry, and biology and pave the way for broader usage in clinical application.

### Cholera toxin subunit

Cholera, mainly caused by *Vibrio cholerae* (*V. cholerae*) O1 and 139 serotypes, is a highly contagious acute dehydrating diarrheal disease. Four oral cholera vaccines are available in the global market, including Dukoral® (SBL Vaccine AB, Stockholm, Sweden), Shanchol® (Shantha Biotechnics Limited, Bashir Bagh, India), and Euvichol® (EuBiologics, South Korea) [62, 63]. Dukoral® is the first prequalified vaccine meeting World Health Organization (WHO) recommendations and contains inactivated *V. cholerae* and recombinant cholera toxin subunits B (rCTB). Shanchol® and Euvichol® are modified version of Dukoral®, which do not require a buffer. These vaccines were respectively prequalified in 2011 and 2015. EuBiologics changed the presentation of the vaccine from conventional glass vials to plastic tubes as Euvichol-Plus®, which is prequalified by WHO in 2017 [64].

Cholera toxin (CT) is the main virulence factor of *V. cholerae* that also possesses strong mucosal immunogenic properties. The adjuvant activity appears to be closely linked to its adenosine diphosphate (ADP)-ribosylating action. However, the toxicity of CT makes it unsuitable for use, necessitating the removal of toxicity while maintaining adjuvanticity. The two main subunits of CT are CTA and CTB. The CTA subunit is responsible for the disease phenotype, while CTB acts as a vector for CTA delivery to target cells. CTA is a 28kD subunit containing two major domains: CTA1, the active part of the toxin, and CTA2, which acts as an anchor for the CTB subunit. CTB subunit is composed of about 55 kD homologous pentamer structure that binds with high affinity to GM1 ganglioside on mammalian cells [65]. CTA can improve the body’s local and systemic immunity to antigens, but its strong toxicity can cause serious diarrhea reactions. CTB eliminates the toxicity of CT while retaining strong immunogenicity and adjuvanticity, making it a research hotspot [66]. Coupling antigens to CTB induces a much stronger response via the oral administration route [67]. A common form is to fuse CTB recombinantly to antigens. In addition, CTB can be co-administered with antigens directly or through chemical coupling. CTB can be used as an adjuvant against infection with the amoeba *Naegleria flowleri*, HIV, *Plasmodium yoelii*, and *Heliobacter pylori* infection in the animal model [68–71]. CTB plays an important role in immune regulation mainly by regulating T cell response, playing an important role in cellular immunity and antibody production. The type of immune response induced by CTB as an adjuvant has not yet been determined, with the Th2 type as the main result in many studies, and the Th1 type as the main result in some studies.

Mucosal administration of relevant antigens conjugated to CTB induces oral tolerance with exceptionally high efficiency, providing a promising approach to prevent or treat allergic or autoimmune disorders [72]. CTB dramatically increases mucosal antigen uptake and presentation by different APCs through binding to a ganglioside, making most B cells effective APCs irrespective of their antibody specificity. Meanwhile, CTB mediates stimulation of transforming growth factor-β (TGF-β) and IL-10 production while inhibiting IL-6 formation, explaining the dramatic potentiation of oral tolerance by mucosal antigens presented with CTB [66]. Behcet’s disease (BD) patients were treated with oral tolerance induction by HSP60 p336-351/CTB conjugate in phase I/phase II clinical study and the promising findings give hope to this strategy with antigen/CTB conjugate in patients with BD and other autoimmune or inflammatory disorders [73]. The immunological and toxicological impacts of CTB administration in different host require further investigation. The capacity of CTB to induce potent mucosal immune responses and the development of efficient CTB/CTB-antigen expression strategies make it applicable in multiple areas, including vaccine development and prevention or treatment of allergic or autoimmune disorders. These findings suggest that CTB-based therapies need further exploration and exploitation.

## Bacterial extracts and preparations

### Bacterial outer membrane vesicles (OMVs)

In 1967, Chatterjee and Das reported that *V. cholerae* could secret particles in the form of bulging out and pinching-off of portions [74]. Membrane vesicles (MVs), usually referred to as OMVs, were first found to be produced through controlled blebbing of the outer membrane of Gram-negative bacteria. Certain Gram-positive bacteria have also been shown to produce similar vesicles [75]. MVs can differ in their structure and composition, including OMVs, outer-inner membrane vesicles (OIMVs), cytoplasmic membrane vesicles (CMVs), and tube-shaped membranous structures (TSMSs).

OMVs are spherical buds of outer double-membrane-like structures, approximately 20 to 400 nm in diameter, filled with periplasmic content, and commonly produced by Gram-negative bacteria [76]. OMVs can be considered bacterial ‘sample packs’, containing much of the biological content found within the parent bacterium but in a non-replicative form [77]. All types of Gram-negative bacteria produce OMVs in a variety of environments, including biofilms and within mammalian hosts [78, 79]. OMVs are nano-sized spherical vehicles and contain multiple parent bacterial-derived components, such as lipopolysaccharide, lipoprotein, DNA, RNA, peptidoglycan, and others. Based on their structures, OMVs have been developed for biomedical applications such as vaccines, adjuvants, and drug delivery vehicles [80]. OMVs are reported to have important biological roles in pathogenesis and intercellular interactions and the structure and composition of OMVs are the basis of their function. An important feature of OMVs is that the proteins associated with them exhibit biological activities. The OMV vaccines from *N. meningitidis* serotype B have been explored since the 1970s and have succeeded in the outbreak of meningococcal group B (MenB)-caused meningitis in Cuba, Norway, and New Zealand have proven the concept of their efficacy [81, 82].

OMVs consist of properties including non-replicating properties, nano-particles, pathogen-like properties, and multiple PAMPs, which have increased the appeal of OMVs as ideal adjuvant candidates. Immunized OMVs with model antigens like ovalbumin or specific pathogen antigens like hepatitis B virus surface antigen have displayed strong adjuvant activity [83, 84]. Integrating antigens into OMVs is another approach to improve the immunization efficacy of antigens of interest. Glycosylated OMVs displaying pathogen glycotopes have been reported to trigger bactericidal activities via capsular polysaccharide or LPS-specific antibodies [85, 86]. An adjuvant formulation of MenB OMV containing mite allergens from Dermatophagoides siboney is undergoing a phase I clinical trial [87]. The safety of native OMVs from *N. meningitidis* has been confirmed in humans, and the MenB vaccine product Bexsero OMVs has been approved by European Medicines Agency (EMA) and US Food and Drug Administration (FDA), further validating the safety of OMVs used as an adjuvant [88]. The mechanism of the adjuvant activities of OMVs remains elusive. PAMPs present in OMVs recognized by PRRs promote activation and maturation of APCs, which express major histocompatibility complex class II (MHC-II) as well as B7 proteins, a necessary co-stimulatory molecule for T cell activation [5]. Engineered OMVs of *Salmonella typhimurium* have been discovered to express membrane-bound antigen program APCs for cross-presentation to CD8^+^ T cells via the MyD88 signaling pathway [89]. Furthermore, it has been proposed that OMVs influence immune response by the “geographic concept”, involving increased antigen uptake and translocation to lymph nodes by APCs.

OMVs have also been studied in the field of cancer immunotherapy and drug delivery vehicles. Yong Song Gho et al. reported bacterial OMVs could effectively induce long-term IFN-γ-dependent antitumor immune responses without notable adverse effects [90]. With the development of genetic engineering, OMVs can be engineered with enhanced tumor-targeting ability and antitumor immune response. In addition, drug delivery vesicle is a promising novel approach for OMVs, as OMVs could be decorated with targeting ligands and accumulated in tumors. Sangyong Jon et al. utilized the bioengineered *E. coli* OMVs to deliver small interfering RNA (siRNA) to target and kill cancer cells [91]. In conclusion, bacterial OMVs hold potential as promising vaccine platforms, delivery carriers, and immune adjuvants. However, further bioengineering and optimization of OMVs is required to reduce endogenous proteins and endotoxin activities, improve productivity, and enhance exogenous antigen loading. Therefore, extensive research efforts aimed at understanding the underlying mechanisms of OMV production and optimizing their design will be essential for advancing their use and implementation.

### Corynebacterium parvum (*C. Parvum*)

*C. parvum*, recently renamed as *Cutibacterium acnes*, is a commensal Gram-positive bacterium that has been shown to be an effective adjuvant when added to normal vaccines, enhancing both soluble and cell-mediated immune responses [92]. Halpern first reported the phagocytic activity of the reticuloendothelial system by *C. parvum* [93]. Series studies in vitro/in vivo of animal models reported the peculiar immunomodulating roles of *C. parvum* against a wide variety of virus infections, such as hepatitis B, herpes simplex virus-1 (HSV-1), influenza virus, rabies virus, human immunodeficiency virus (HIV), and so on [94–97]. Liu et al. reported that *C. parvum* can induce peripheral blood monocyte differentiation into CD209^+^ macrophages and CD1b^+^ DCs in vitro. CD209^+^ cells are more effective in binding and phagocytosis of microbial pathogens, while CD1b^+^ cells with an immature DC phenotype are efficient antigen-presenting cells to adaptive T cells response [98]. In addition, both CD209^+^ and CD1b^+^ cell populations secret human IL-12 or IL-23 to modulate the adaptive Th1 response [99]. The host receptors involved in the immunostimulatory process of *C. parvum* remain unknown, due to the host receptors recognize highly conserved microbial structures, which are potential candidates for its immunostimulatory effect. TLR2, TLR9, and MyD88 are related to the immunostimulatory effects of *C. parvum* on both innate and adaptive immunity. Meanwhile, *C. parvum* can activate macrophages, enhance the activity of NK cells, enhance the ability of B cells to produce antibodies, and induce the production of cytokines such as IFN, TNF, and interleukin. However, *C. parvum* may enhance the toxicity of LPS endotoxin through the induction of TNF by immune-competent cells [100].

The very first clinical use of *C. parvum* as a drug (Coryparv, by the International Wellcome Burroughs Company) was in the oncological setting because of its direct anticancer properties, resulting in prolonged survival and better quality of life. However, the registration of Coryparv, a drug containing *C. parvum*, expired in Europe [101]. The wide spectrum antiviral activity of *C. parvum* is supposedly due to activity potentiating monocytes, and NK cells with regards to their number and function, and interferon gene activation, which is the first barrier to virus invasion [92]. During the coronavirus disease 2019 (COVID-19) pandemic, the superior activities of *C. parvum* in promote innate immunity and modulate adaptive immunity have attracted researchers’ attention. Palmieri et al. confirmed that *C. parvum* is safe and effective in supporting immunocompromised patients during epidemic or pandemic events when the risk of infection and complications is increased, including COVID-19 infection [101]. The antiviral properties suggest its potential and effective use to fortify fragile patients. C. parvum may be used to prevent COVID-19 and other virus infections by blocking the virus entrance and interrupting early symptoms in the respiratory system [99]. When sudden pandemic occurs, the inactivated *C. parvum* is promising to be used as an innate immunity trigger, helping to quick and effectively get rid of virus.

### The BCG and BCG cell wall skeleton (BCG-CWS)

Tuberculosis infection is characterized by a complex immunologic response, leading to a unique host-pathogen interaction. BCG is an attenuated strain of *M bovis*, isolated by and named after Calmette and Guérin in Lille, France, which is used as vaccines against tuberculosis [102]. Benjamin Weill-Halle and Turpin gave a dose of BCG to the infant by oral route in 1921, showing no undesirable sequelae [103]. BCG vaccination has been shown to be safe and effective, with several vaccines in use today, such as products of Pasteur-Merieux-Connaught, the Danish Statens Serum Institute, Evans Medeva, and the Japan BCG Laboratory in Tokyo. BCG vaccination has been associated with a reduction in infant mortality due to protection against respiratory tract infections and neonatal sepsis [104].

Experiments with BCG as cancer treatment began and in 1976 Morales published the first successful clinical results of nine patients with superficial bladder tumors treated with intravesical BCG and the intravesical instillation of BCG for treating bladder cancer is a powerful cancer immunotherapy [105]. BCG is available in the clinic (Immunobladder®) and it obtains an overwhelmingly high therapeutic effect that indicates it will be used in the treatment of cancers other than bladder cancer [106]. BCG has potent immunological effects, including augmentation of the antibody response, increased delayed hypersensitivity responses to a variety of antigens, increased activity of peritoneal macrophages, and increased clearance of bacteria [107]. In the current, COVID-19 pandemic situation, numerous vaccines against emerging and re-emerging infections have limited immunity and a prior BCG vaccination is also implicated with better clinical outcomes from COVID-19 [104, 108]. The COVID-19 pandemic has renewed researchers’ interest in this old vaccine and the increasing scientific investigations will provide new ideas for the understanding of immunology.

The BCG-CWS is the central immune activator and potent substitute of BCG, with a basic structure composed of mycolic acid, arabinogalactan, and peptidoglycan. BCG-CWS can act as ligands for TLR2 and TLR4, leading to DCs maturation and secretion of TNF-α, IL-6, and IL-12 p40 [109]. BCG-CWS can also be recognized by C-type lectin receptors, such as macrophage-inducible C-type lectin and dectin-2, via trehalose dimycolate and mannose-capped lipoarabinomannan [109–111]. However, BCG-CWS is insoluble in both aqueous and organic solvents, and the use of mineral oil and Tween 80 to form an oil-in-water (O/W) emulsion of BCG-CWS applied to animals and humans, leads to limited routes of administration and strong local inflammation [112, 113]. Although BCG-CWS immunotherapy has been extensively studied in clinical trials, there are still issues to be solved. For example, BCG-CWS immunotherapy seemed to improve survival after resection of non-small cell lung cancer (NSCLC), especially locally advanced NSCLC [114]. Kim et al. investigated BCG-CWS adjuvant effects on influenza vaccine efficacy in both infant and old-aged mice [115]. Masuda and Nakamura explored a kind of water-dispersible nanoparticle formulation of BCG-CWS that could be applied as a systemically injected adjuvant for cancer immunotherapy [102, 116].

BCG-CWS exhibits potent immunomodulatory activities, however, further exploration of its underlying molecular mechanisms and advancements in technology are necessary to enable its wider application across multiple fields. As such, extensive research efforts aimed at understanding the molecular basis of BCG-CWS-induced immune modulation, as well as developing new technologies for its production and application, are needed to facilitate broader usage of this potent immunostimulant.

### Hemolytic streptococcus preparation OK-432

OK-432 is a bacterial-origin immunostimulant that is a lyophilized streptococcal preparation from a low-virulence strain of *Streptococcus pyogenes* incubated with penicillin. It is utilized as an immunotherapeutic agent in cancer treatment via the induction of Th1-type cytokines [117]. OK-432 was first reported to have potent antitumor activity in the early 1970’s by Sakurai and his team members [118]. OK-432 induces Th-1 cytokines, such as IFN-γ and IL-12, as well as shifting the Th-1/Th-2 balance to a Th-1 dominant state [119]. OK-432 can activate NK cells, macrophages, polymorphonuclear cells, and CD4^+^T cells [120–122].

In Japan, OK-432 has been used for more than two decades as a good manufacturing product (GMP)-grade immune-potentiating agent [123]. Preclinical and clinical trials have investigated the use of OK-432 for cancer treatment and lymphangiomas. It is reported that intratumoral administration of immature DCs in combination with OK-432 accelerated the antitumor effect of immature DCs in animal models [124]. A phase I trial of preoperative intratumoral immature DCs injection together with OK-432 is considered to be a safe and feasible procedure for neo-adjuvant immunotherapy for pancreatic cancer [125]. In addition, intralesional injection of OK-432 is an effective treatment and should be considered the primary method of treatment for lymphangiomas, which was employed to treat 64 lymphangiomas between 1986 and 1992 [126]. A phase II clinical trial at 27 U.S. academic medical centers reported by Mark demonstrated that OK-432 immunotherapy is an effective, safe, and simple treatment option for the management of macrocystic cervicofacial lymphatic malformations [127]. Intralesional injection of OK-432 is a safe and effective therapy for lymphangioma and OK-432 could activate white blood cells to secret cytokines including tumor necrotic factor, which increased endothelial permeability and then accelerated lymph drainage and increased lymph flow leading to shrinkage of the cystic spaces [128]. The OK-432 was then exploited to treat plunging ranula, similar to a cystic lymphangioma, suggesting it was a safe and effective therapy resulting in a complete or a significant decrease in the volume of cysts [129, 130]. The efficacy of OK-432 treatment is more often confirmed in antitumor immunity for cancer treatment, such as thyroglossal duct cysts, auricular hematomas, pneumothorax, pediatric solid tumors, advanced ovarian cancer, and so on [131–133]. It is reported that OK-432 (also called sapylin) could improve the immunity of patients with thoracic malignancies significantly in the early postoperative period [134].

In summary, OK-432 shows promise as an immunotherapeutic agent in cancer treatment. Its ability to induce Th1-type cytokines and activate immune cells makes it a potentially effective treatment option for a range of medical conditions. Bacterial therapy is often studied in cancer treatments research indicates the immunomodulatory potential of bacterial preparation, and other bacterial strains, including Bifidobacteria, Salmonella, and Clostridia species, are potential candidates for tumor treatment. Detailed evaluation of the toxicity associated with bacterial preparations necessitates long-term studies. However, innovation approaches, such as the engineering of bacterial strains, hold potential for promoting the expansion of bacterial therapy development and tumor immunity. Meanwhile, the exploration and revelation of the mechanism underlying its immunotherapeutic effect associated with bacterial preparations will facilitate their broader application across multiple fields.

## Conclusions and future perspective

Emerging infectious diseases and heightened risk of cancer are huge challenges for public health in current society. Current vaccines have demonstrated low cross protection against pathogen isolates, resulting in undesirable immune responses that fail to clear them. Therefore, the use of immunostimulants, particularly those targeting innate immunity, offers promising strategies to improve vaccine effectiveness.

**Fig. 5:**
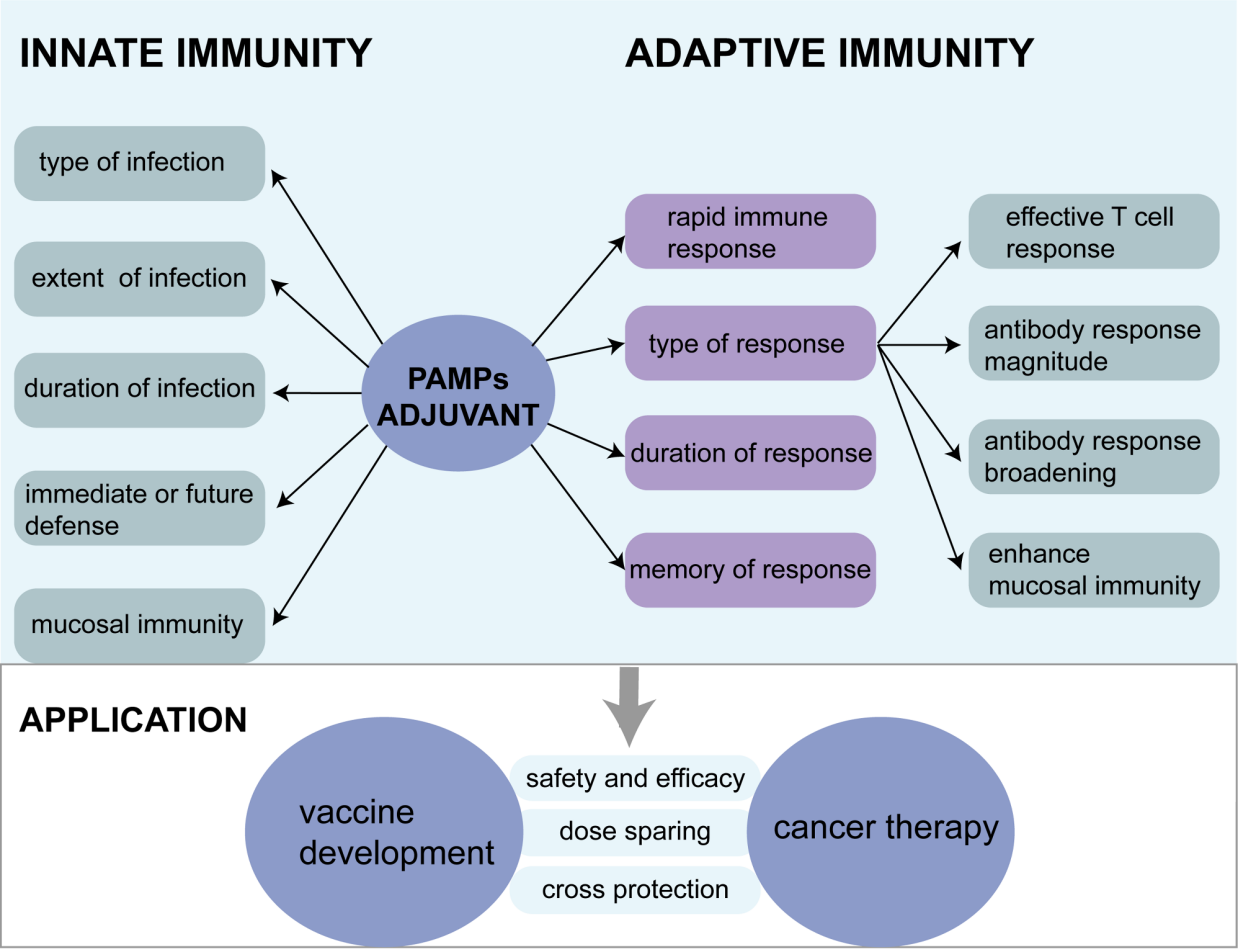
Advantages and potential applications of bacterial origin immunostimulants. Bacterial component or bacterial component-mimicking compounds can be recognized by PRRs and activate both innate and adaptive immune responses. PAMPs-PRRs rapidly mediate innate immune response and determine the type, extent, duration of infection, and the requirement for immediate or future defense. Meanwhile, some PAMPs can activate mucosal immunity, which is significant for pathogen trafficking. Interaction between bacterial component or bacterial component-mimicking compounds and PRRs can promote a more efficient adaptive immune response, including more rapid, magnitude, broadening, and long-time antibody response and IgA secretion. Research and development of appropriate bacterial-origin immunostimulants may be filled the gaps both in vaccine production and cancer therapy. PRR, Pattern recognition receptor; PAMP, pathogen-associated molecular pattern

The discovery of adjuvants has historically been guided by empirical observations, with aluminum salts being the sole adjuvant approved by the FDA for extensive utilization spanning several decades [135]. Nevertheless, aluminum salts exhibit limited adjuvanticity for certain antigens, such as those associated with influenza [136]. In response to these limitations, immunostimulants have emerged as a critical component for addressing the unmet clinical needs in vaccine development and therapeutic protocols. The burgeoning use of highly purified recombinant antigens also necessitates the advancement of immunostimulants. The advantages and potential applications of bacterial-origin immunostimulants are described in Fig. 5. In addition, bacterial-origin immunostimulants have their respective characteristics, advantages, and even limitations (Table 2). Clarifying these properties will also help guide their design application. With the advancements in our comprehension of immunological mechanisms, coupled with technological innovations, the field of adjuvant development has transitioned from a reliance on chance discoveries to strategic, rational design.

**Table 2:** Main characteristics of the representative of bacterial original immunostimulants

Immunosimulants	Additional information	Immune responses	The merits	The drawbacks	(Potential) applications
**MPL**	Detoxified TLR4 ligand	TLR4-TRIF signaling; TNF-α, IL-6, IFN-γ production; Th1 response	induce high and persistent-specific antibody responses	Contains several different MPL species that vary in length and degree/type of fatty acid acylation; MPL manufacturing process from LPS is complexity; hydrophobic nature; local adverse events (pain, erythema and swelling)	A component of AS04 in HBV vaccine Fendrix, and HPV vaccine Cervarix, a component of AS01 in varicella zoster vaccine Shingrix; vaccines targeting infectious diseases; vaccines targeting allergens; vaccines targeting cancers
**Bacterial lipoproteins/lipopeptides**	Lipidated N-terminus of bacterial lipoproteins behave as potent adjuvant; Representative synthetic lipopeptide: Pam_n_Cys analogues Macrophage-activating Lipopeptide-2 (MALP-2) from *Mycoplasma fermentans* was synthesized as Pam_2_Cys; Pam_3_Cys ligand is derived from the N-terminal motif of *Braun’s lipoprotein*, a murein lipoprotein found in the cell walls of Gram-negative bacteria	PamnCys analogues activate TLR2/TLR1 or TLR2/TLR6 signaling; IFN-γ and TNF-α production; Th1, Th17 response	Potential to be a self-adjuvating vaccine; ease of design and synthesis small molecule lipopeptide	Potential adverse side effect due to the antigenic domain of a bacterial lipoprotein; structures of full-length bacterial lipoproteins are few and far between	self-or auto-adjuvating vaccine candidates
**Flagellin**	Co-mixed with antigens; Chimeric expression or co-display of flagellin and foreign antigens in live bacterial strains; Recombinant proteins consisting of heterologous antigens fused to flagellin	TLR5 signaling with TNF-α, IL-10, IL-8, IFN-γ production; NLRC4/NAIP5 signaling with IL-1β production; Th1 and Th2 response	Excellent adjuvant activity (low dose); modifiable; flexible and efficient for antigen delivery	Anti-flagellin antibody could interfere with its adjuvant activity; potential low immune response to antigens fused to flagellin	Mucosal vaccines; antigen delivery vehicle; immunotherapy
**Bacterial CpG oligonucleotides**	synthetic oligodeoxynucleotides containing a CpG ODN mimicking bacterial DNA	TLR9 and B-cell receptor or Src kinase family signaling; TNF-α, IL-1β, IL-1α, IL-6 production; Th1 response	Mucosal immune responses; boost immunity in groups with reduced immune function; directly activate human B cells	upregulation of indoleamine 2,3-dioxigenase expression in DCs, a well-described mediator of immunological tolerance; frequency and severity of local adverse events (injection site reactions such as pain, swelling, induration, pruritus, and erythema) and systemic symptoms (including flu-like symptoms) were elevated; increased host susceptibility to autoimmune disease or a predisposition to toxic shock	CpG1018 in hepatitis B vaccine (approved by FDA in 2017); Vaccines targeting allergens; Vaccines targeting cancer; Adjuvant for DNA vaccine; Applications in enhancing the immune responses of vaccines in immunocompromised populations
**Cholera toxin subunite**	CTB forms a ring-like structure composed of five CTB monomers; Recombinant proteins consisting of heterologous antigens fused to CTB; Co-mixed with antigens	GM1 receptor mediating uptake; IL-4, IL-10, IFN-γ, IL-1β production; Th2 and Th1 response	mucosal immune responses; stability; ease to express CTB; modifiable; recognition receptor GM1 expressed in B cells	Potential low expression level with fusion antigen to CTB	Mucosal adjuvant; antigen delivery vehicle; Adjuvant for DNA vaccine; Vaccines targeting allergens and autoimmune disorders
**Bacterial OMVs**	OMVs display a complex array of PAMPs, such as LPS, flagellin, and peptidoglycan, in their native conformation; Engineered to express antigenic proteins of interest or modulate toxicity and adjuvating effect, or target delivery	OMV-associated PAMPs contributing to innate immune response in various cell types; Th1 and Th2 response	Modifiable; nanoparticles	Toxicity of LPS and other OMPs; high cost for manufacture	OMV-based vaccine against meningococcus MenB (4CMenB, Bexsero; GSK); Antigen-delivering platform; immunotherapy; mucosal adjuvant
***Corynebacterium parvum***	unique aspecific antiviral properties	interferon family, IL-8, IL-1b, TNF-α, IL-12, IL-15, GM-CSF induction; TLR2, TLR9, and MyD88 signaling; Th1 response	Strong macrophage activator; increase lymphocyte count (helper and killer lymphocytes)	Non-specific activation; Side effect like fever, malaise, and local reactions at the site of injection; potential for causing disease; enhance the toxicity of LPS endotoxin	antiviral activity during a pandemic emergency; cancer treatment; applications in enhancing the immune responses of certain vaccines
**BCG-CWS**	a basic structure composed of mycolic acid, arabinogalactan, and peptidoglycan	TLR2 and TLR4, C-type lectin receptors signaling; TNF-α, IL-6, and IL-12 p40 production; Th1 response	long-lasting adaptive immune response	Insoluble in both aqueous and organic solvents; limited routes of administration and strong local inflammation	cancer treatment
**OK-432**	A streptococcal preparation	Activating various aspects of the immune response; IL-6, IL-8, IFN-γ, TNF-α, IL-12 p70 production; Th1 response	Potent and multifaced immune activation; a potent maturation factor for Mo-DCs	Side effect like fever, chill, and other flu-like symptoms; not tumor-specific	A cancer immunotherapy drug in Japan; Cancer treatment including lymphangiomas, pleural effusion due to lung cancer; otolaryngological cystic diseases therapy

MPL, Monophosphoryl lipid A; OMVs, outer membrane vesicles; BCG-CWS, BCG cell wall skeleton; CTB, Cholera toxin subunit B; GM1, Monosialotetrahexosylganglioside; NLRC4, Nod-like receptor protein 4; NAIP5, Nucleotide-binding domain leucine-rich repeat family, apoptosis inhibitory protein 5; Mo-DCs, monocyte-derived dendritic cells

The growing recognition of innate immunity’s central role in shaping vaccine adjuvant activity has emerged as a critical area of investigation within the field of vaccinology [137]. Bacterial active molecules are natural agonists of PRRs like TLRs, which can significantly boost the host immune system, making them great potential for anti-infection and anti-tumor. MPL, a TLR agonist, led the way as the first of its kind to be approved as part of the adjuvant system AS04 in the Cervarix vaccine, which received licensure in 2009 [138]. Subsequently, the implementation of CpG 1018 in the HBV, approved in 2017, underscored the clinical relevance of TLR agonists [139]. These milestones have spurred further research into the mechanisms and applications of TLR agonists as vaccine adjuvants. Bacterial immunostimulants initiate an immune cascade by activating stromal and resident immune cells at the site of infection and recruiting additional immune cells to the fray. Engagement of these immunostimulants with PRRs assists in the mobilization and activation of various innate cell types, most notably APCs like monocytes and dendritic cells. These activated APCs, armed with antigens, journey to the draining lymph nodes where they orchestrate the antigen presentation to T cells, thereby catalyzing the adaptive immune response. The specificity of bacterial-derived immunostimulants for PRRs enhances APC activation, as evidenced by increased expression of co-stimulatory molecules, which in turn leads to more efficient antigen presentation to CD4^+^ T cells. An elevated number of robust CD4^+^ T cells can better guide B-cell differentiation, ensuing a surge in antigen-specific memory B-cell populations [140]. With the in-depth study of innate immune mechanism, TLRs agonists as new molecular adjuvants have shown good application prospects and deserve further study.

Tumor-specific immunotherapy represents a frontier in cancer treatment, yet presents significant challenges due to the often weak immunogenicity of tumor antigens, attributable in part to their resemblance to self-antigens. The immune evasion tactics of tumors, such as the suppression of DC maturation and function, further complicate the therapeutic landscape. Immunostimulants are thus becoming an indispensable element of tumor vaccine strategies [141]. For cancer therapy, the ideal vaccine adjuvant would consistently provoke a type 1-polarized, cellular immune response, rather than a predominantly type 2-polarized or antibody-mediated humoral response. To date, efforts to enhance cellular responses through vaccination have not produced significant advances. Moreover, MPL has demonstrated the ability to elicit strong Th1-polarized and cellular immune responses. Similarly, CpG oligodeoxynucleotides have shown efficacy in stimulating robust Th1 and cytotoxic T-cell responses. The intrinsic characteristics of MPL and CpG position them as promising candidates for anti-tumor therapy. Additional research into the molecular workings of various bacterial-origin immunostimulants is critical to enabling more innovative vaccine design. The modulation of the cytokine network by bacterial-origin immunostimulants is a key mechanism through which these agents activate the immune system [142]. Cytokines are vital regulators of both innate and adaptive immunity; thus, immunostimulatory cytokines, such as IL-2, IFN-γ, IL-12, and granulocyte-macrophage colony-stimulating factor (GM-CSF), are promising candidates for inclusion in vaccines. Nevertheless, the therapeutic efficacy of cytokines as adjuvants has been inconsistent, perhaps owing to variabilities in dosage and administration regimens [143]. The use of cytokines also brings concerns over toxicity, particularly when high concentrations breach the systemic circulation, as well as limitations arising from their pleiotropic nature and brief half-life—both factors implicated in the induction of toxicity. Consequently, several strategies have been employed to circumvent these complications, including the encapsulation of cytokines within liposomes and the co-delivery of cytokine-expressing vectors alongside DNA vaccines.

Addressing safety and regulatory concerns is paramount when introducing novel immunostimulants into vaccine formulations. To date, bacterial-origin immunostimulants utilized in commercial or clinical settings have exhibited a relatively acceptable safety profile. However, as these immunostimulants often derive from bacterial virulence factors, there is an inherent risk of triggering overactive immune responses via PRRs, potentially leading to excessive or uncontrolled inflammation. This double-edged sword of bacterial-derived immunostimulants underscores the complexity of developing safe yet effective adjuvants. Mitigating toxicity while preserving or enhancing immune response is a critical focus. Advancements in our understanding of PRR signaling and immune activation pathways empower researchers to rationally design bacterial-derived molecules, tempering their ability to over-stimulate the immune system and thereby minimizing harmful outcomes. The creation of synthetic analogs offers a strategic pathway towards optimizing these molecules, achieving greater efficacy with reduced toxicity. The targeted delivery of immunostimulants to select immunocompetent cells emerges as a significant strategy to constrain their systemic spread and prevent excessive inflammatory reactions. Integrating such approaches is indicative of the inherently multidisciplinary nature of adjuvant discovery, which straddles the fields of formulation science, immunology, toxicology, and biology, not to mention the business considerations of sourcing, cost, accessibility, and intellectual property. The successful implementation of MPL and CpG 1018 immunostimulants within licensed vaccines acts as both a testament to their clinical utility and a beacon for future adjuvant development.

In recent years, the chemical synthesis, modification, and assembly of bacterial active molecules have become a focal point of research, transforming these molecules into advanced materials. Consequently, there is a recognized need for more in-depth investigation into bacterial PAMPs and their associated pathogenesis. The use of potent adjuvants in vaccination is a promising strategy for the prevention of secondary and concurrent infections, as well as for bolstering overall immune responses [144]. In the backdrop of ongoing pandemic challenges, such as COVID-19, traditional BCG vaccine may serve as a bridge to a specific COVID-19 vaccine and public health implications of a plausible BCG cross-protection from severe COVID-19 are also being discussed [145]. This underscores the compelling capacity of bacterial immune-reactive molecules to activate and strengthen the host immune system.

Many types of adjuvants are currently available for incorporation in vaccines, and their numbers are increasing as many more being described annually. However, only a limited subsets of these adjuvants have undergone evaluation in human subjects, and even smaller number have been incorporated into licensed or commercially available vaccines. When selecting an adjuvant for a particular vaccine, a multitude of factors must be meticulously considered. These considerations extend beyond the adjuvant’s immunomodulatory efficacy, safety profile, and tolerability. They also encompass commercial viability, production feasibility, specific antigenic properties, and the stability of the vaccine formulation, etc. The optimal adjuvant choice is one that achieves a harmonious balance among these varied, yet equally critical, attributes to ensure the ultimate success of the vaccine in both clinical and commercial settings.

Overall, bacterial-origin immunostimulants show great promise for the prevention and therapy of infectious diseases and tumors. It is very meaningful and important to explore and expand our knowledge of bacterial active molecules for immunostimulant development. Further research and development are needed to ensure the safety and efficacy of these immunostimulants, but their potential benefits warrant continued investigation and exploration. Aspiring adjuvant technologies should continually evolve to meet the demands of safety, efficacy, and regulatory approval, ensuring a positive impact on the future landscape of vaccine development.

## Data Availability

Not applicable.

## Abbreviations

PRRs: Pattern recognition receptors
TLRs: Toll-like receptors
APCs: Antigen-presenting cells
PAMPs: Pathogen-associated molecular patterns
LPS: Lipopolysaccharide
MPL: Monophosphoryl lipid A
MyD88: myeloid differentiation 88
TNF: Tumor necrosis factor
AS: Adjuvant System
OMP: Outer membrane protein
*N. meningitidis*: *Neisseria meningitidis*
*B. pertussis*: *Bordetella pertussis*
DCs: Dendritic cells
Pam3CSK4: Pam3-Cys-Ser-Lys4
NLRC4: Nod-like receptor protein 4
STF2: Flagellin phase 2
*M bovis*: *Mycobacterium bovis*
BCG: Bacillus Calmette-Guérin
CpG: Cytosine-phosphate-guanine
CpG ODN: Specific nucleotide fragments containing non-methylated CpG motifs
pDCs: Plasmacytoid dendritic cells
IFN: interferon
*V. cholerae*: *Vibrio cholerae*
CT: Cholera toxin
rCTB: Cholera toxin subunits B
ADP: Adenosine diphosphate
OMVs: Bacterial outer membrane vesicles
MVs: Membrane vesicles
OIMVs: Outer-inner membrane vesicles
CMVs: Cytoplasmic membrane vesicles
TSMSs: Tube-shaped membranous structures
MenB: Meningococcal group B
EMA: European Medicines Agency
FDA: US Food and Drug Administration
MHC-II: Major histocompatibility complex class II
siRNA: small interfering RNA
*C. parvum*: *Corynebacterium parvum*
HSV-1: Herpes simplex virus-1
HIV: Human immunodeficiency virus
COVID-19: Coronavirus disease 2019
BCG-CWS: BCG cell wall skeleton
O/W: Oil-in-water
NSCLC: non-small cell lung cancer
GMP: Good manufacturing product
